# Supplementary material for: Functional proteoform group deconvolution reveals a broader spectrum of ibrutinib off-targets
Source: Nat Commun. 2025 Feb 25;16:1948. doi: 10.1038/s41467-024-54654-8 (PMC11862126; doi:10.1038/s41467-024-54654-8)
Supplement: Supplementary file 1 — Supplementary Info File #1 [file 41467_2024_54654_MOESM1_ESM.pdf]

Supplementary Information for:

Functional proteoform group deconvolution reveals a broader spectrum of ibrutinib off-targets

Isabelle Leo[1], Elena Kunold[1,2], Anastasia Audrey[3], Marianna Tampere[4], Jürgen Eirich[5], Janne Lehtiö[1], Rozbeh Jafari[1]*

[1] Clinical Proteomics Mass Spectrometry, Department of Oncology-Pathology, Karolinska Institutet, Science for Life Laboratory, Solna, Sweden.

[2] Evotec Munich GmbH, München, Germany

[3] Department of Medical Oncology, University Medical Center Groningen, Groningen, Netherlands

[4] Precision Cancer Medicine, Department of Oncology-Pathology, Karolinska Institutet, Science for Life Laboratory, Solna, Sweden

[5] Institute of Plant Biology and Biotechnology, University of Munster, Munster, Germany

Corresponding authors: Correspondence should be addressed to R.J.

Corresponding author email: rozbeh.jafari@ki.se

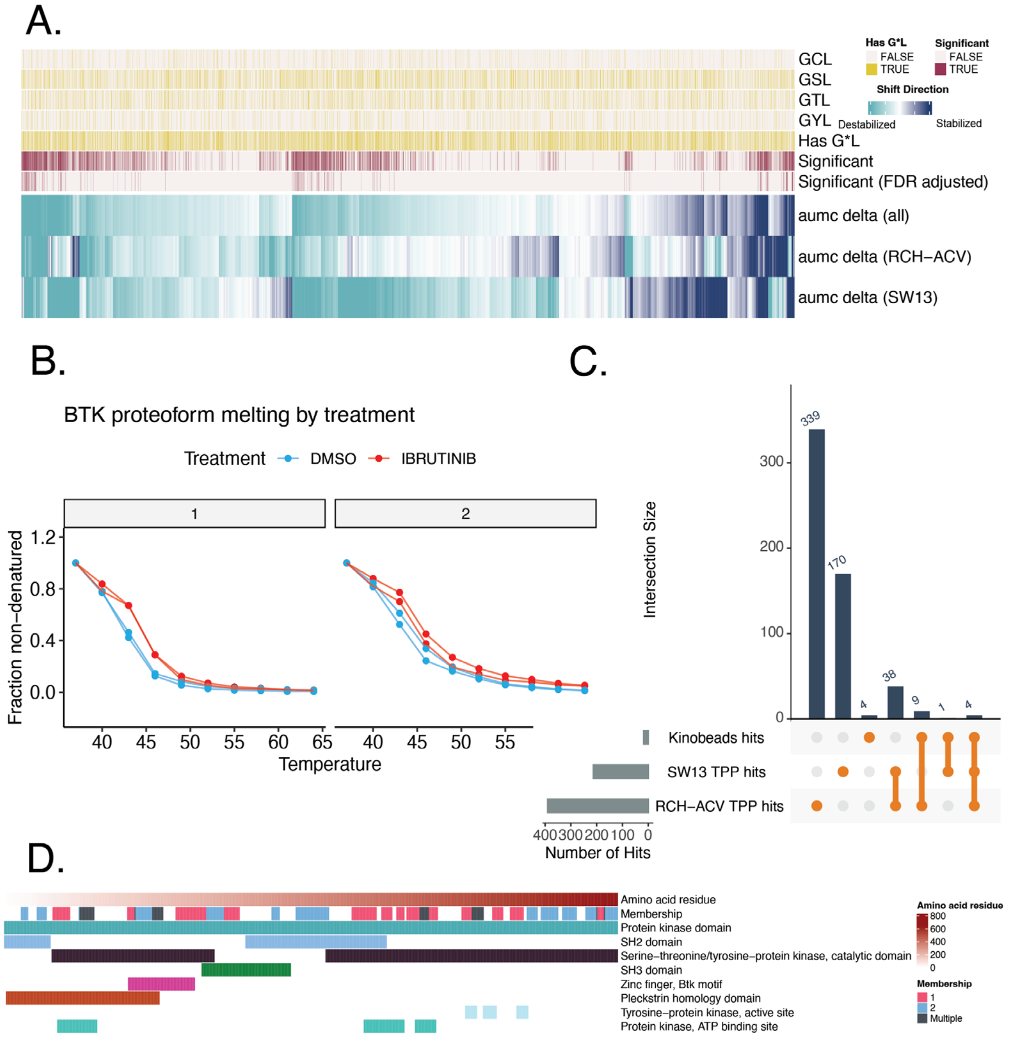

Supplementary Figure 1, Contextual details of ibrutinib binding targets and BTK proteoform groups: A) All proteoform group results for which full melting curve models could be generated are plotted clustered by delta aumc, defined as the aumc of DMSO data with aumc of ibrutinib data subtracted. Highlighted proteoform groups are annotated by presence of GCL, GSL, GTL, or GYL sequences, and by their significance in one or both cell line(s) at a threshold of $p < 0.05$ “Significant” or pAdj < 0.05 “Significant (FDR adjusted)”. **B)** Fraction non-denatured for each BTK proteoform, demonstrating melting of the four replicate curves by treatment. **C)** Detected hits ($p < 0.05$) in each individual cell line stratified by their gene symbol ID, and showing their overlap with the previously identified functional ibrutinib target hits from (*18*). **D)** Peptides mapping to their corresponding locations on the canonical FASTA sequence for BTK, colored by functional proteoform group assignment “Membership” and highlighting regions with domains annotated in the interpro database (*71*)**.**

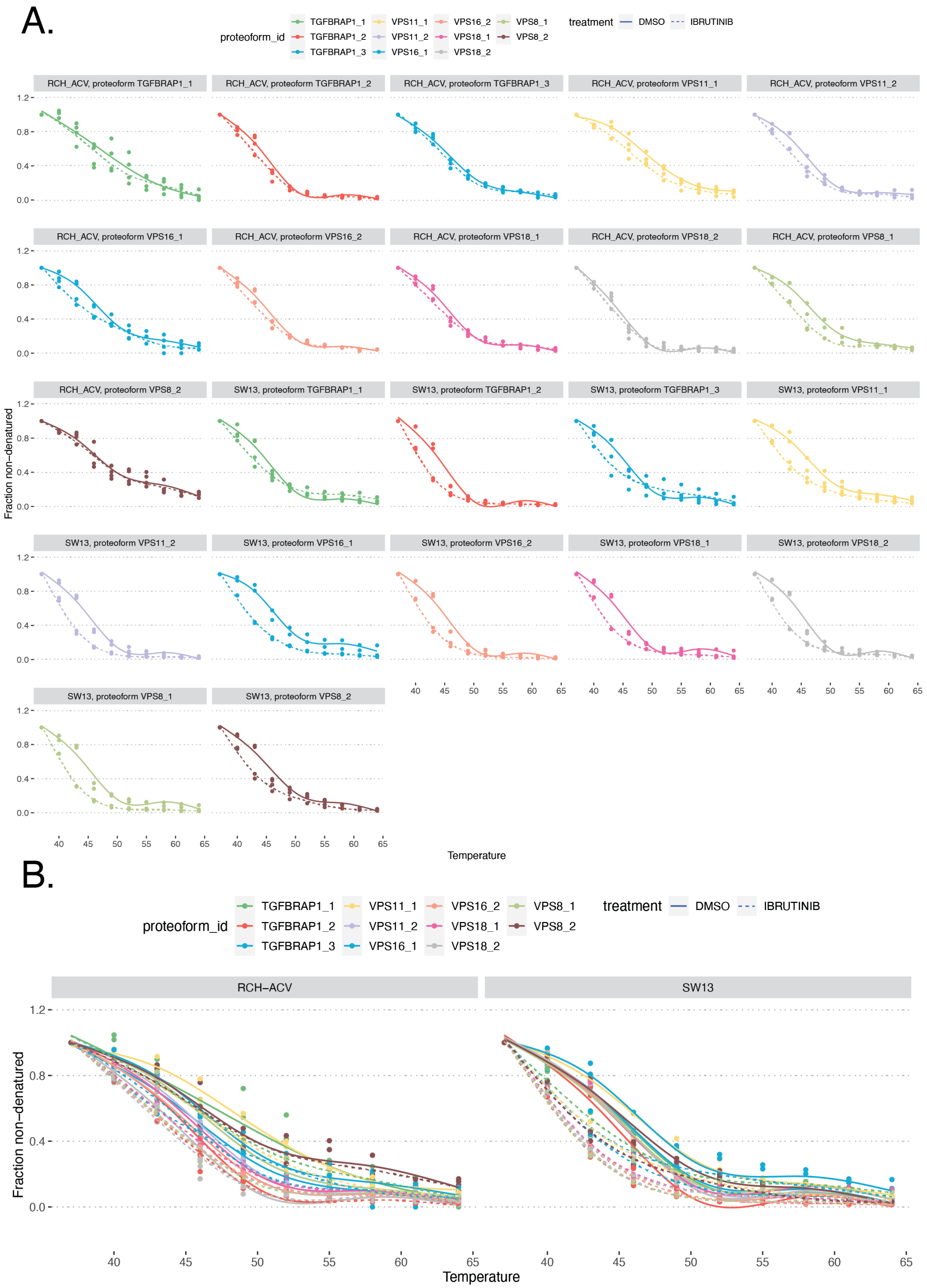

Supplementary Figure 2, Functional proteoform group composition and thermal behavior of the CORVET complex: A) All functional proteoform group melting, for the CORVET complex. **B)** Same data as (A), plotted in one window per cell line, showing detection per cell line.

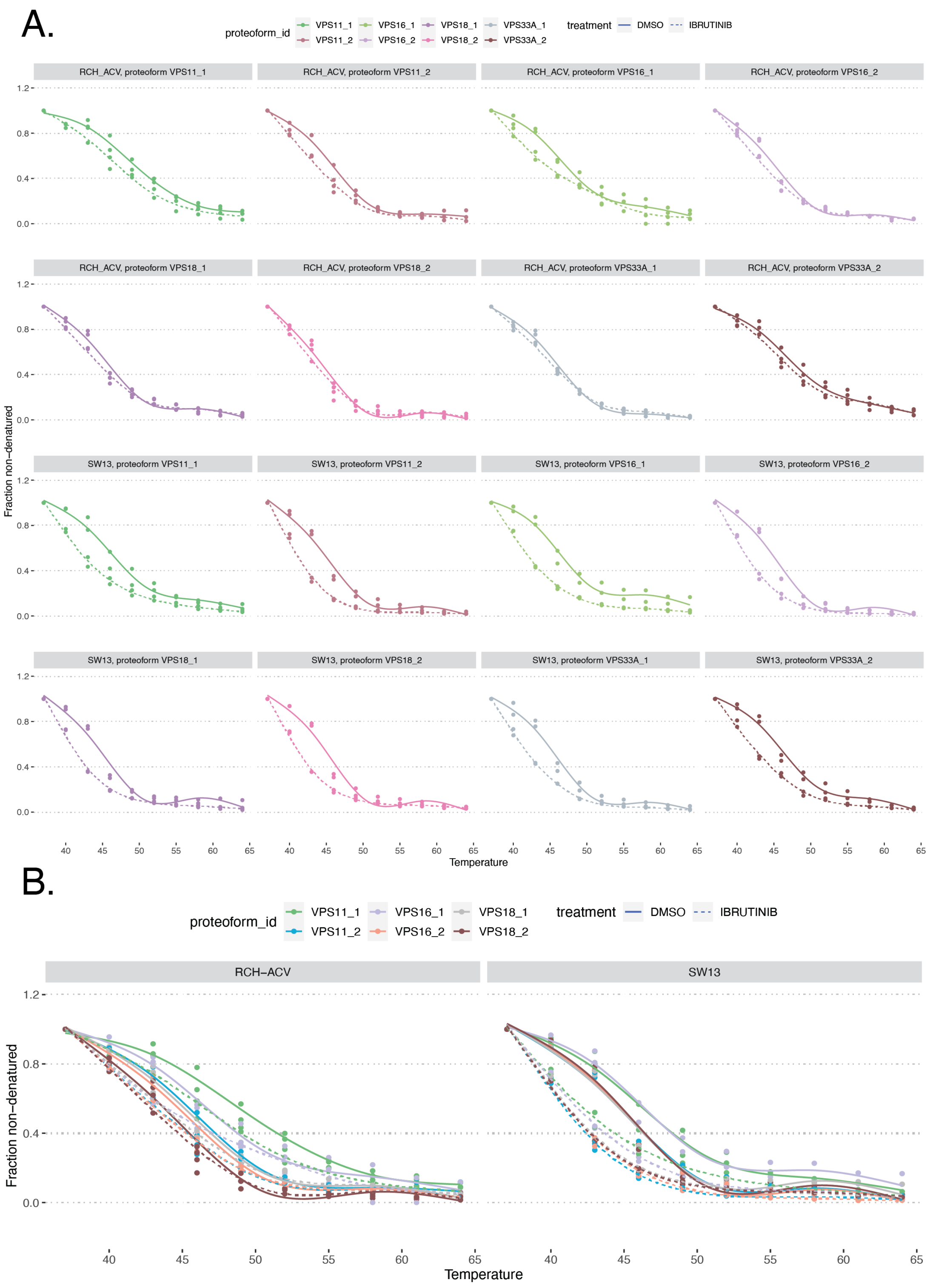

Supplementary Figure 3, Functional proteoform group composition and thermal behavior of the HOPS and class C VPS complex: A) All functional proteoform group melting, for the shared components of the HOPS and class C VPS complexes. **B)** Same data as (A), plotted in one window per cell line, showing detection per cell line.

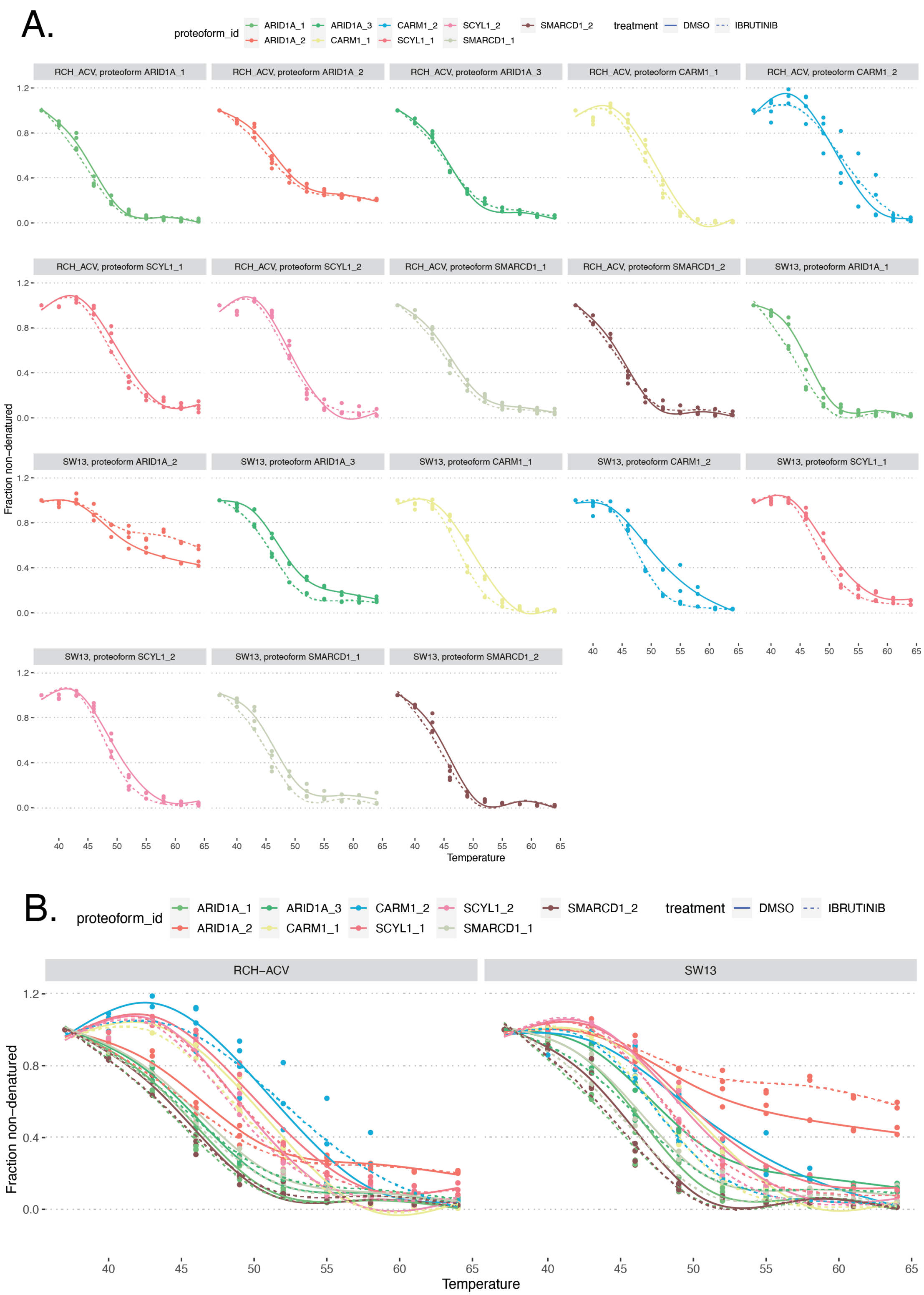

Supplementary Figure 4, Functional proteoform group composition and thermal behavior of the NUMAC complex: A) All functional proteoform group melting, for the NUMAC complex. **B)** Same data as (A), plotted in one window per cell line, showing detection per cell line.

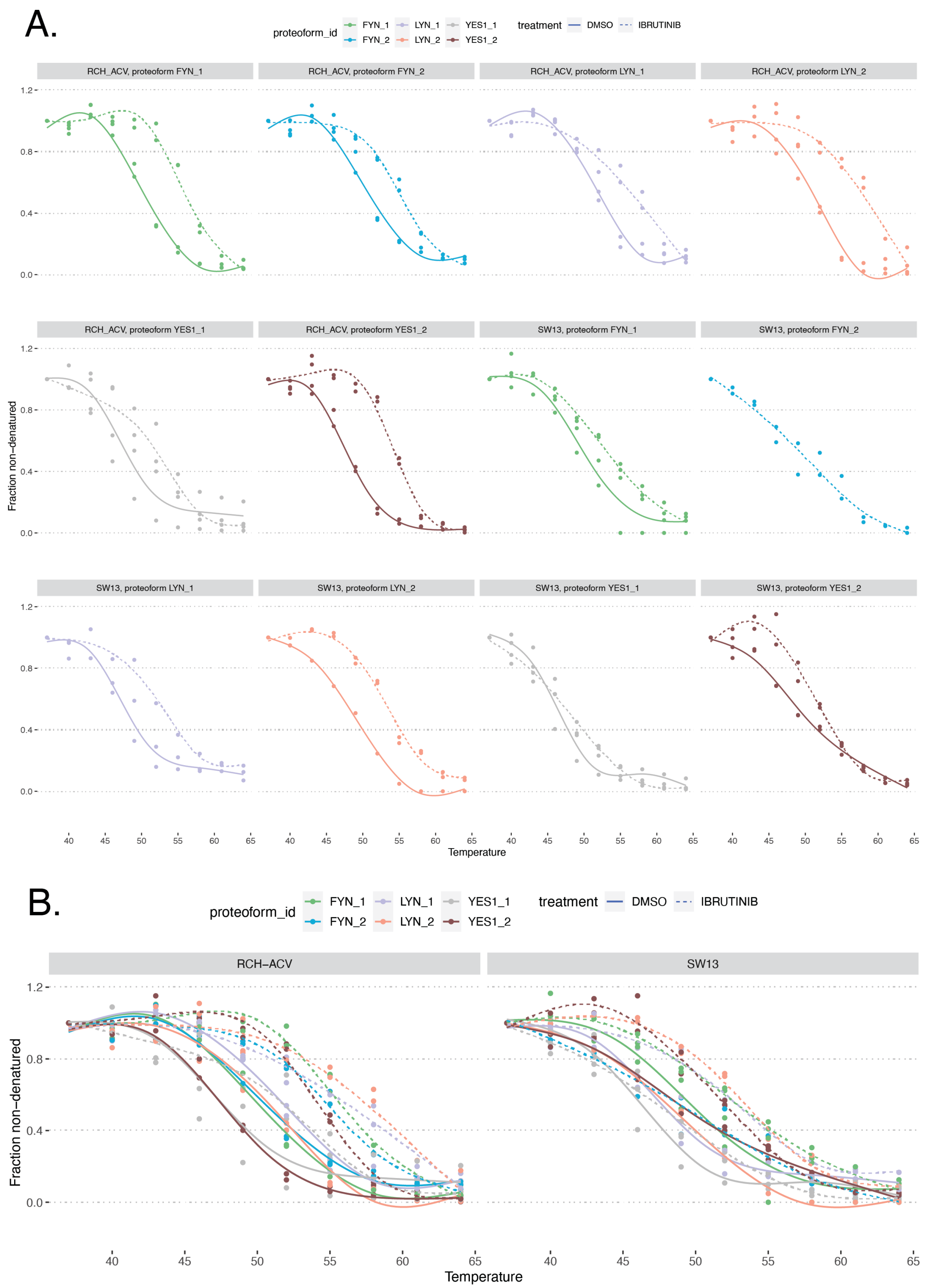

Supplementary Figure 5, Functional proteoform group composition and thermal behavior of the p21(ras)GAP-FYN-LYN-YES complex: A) All functional proteoform group melting, for the p21(ras)GAP-FYN-LYN-YES complex. **B)** Same data as (A), plotted in one window per cell line showing detection per cell line.

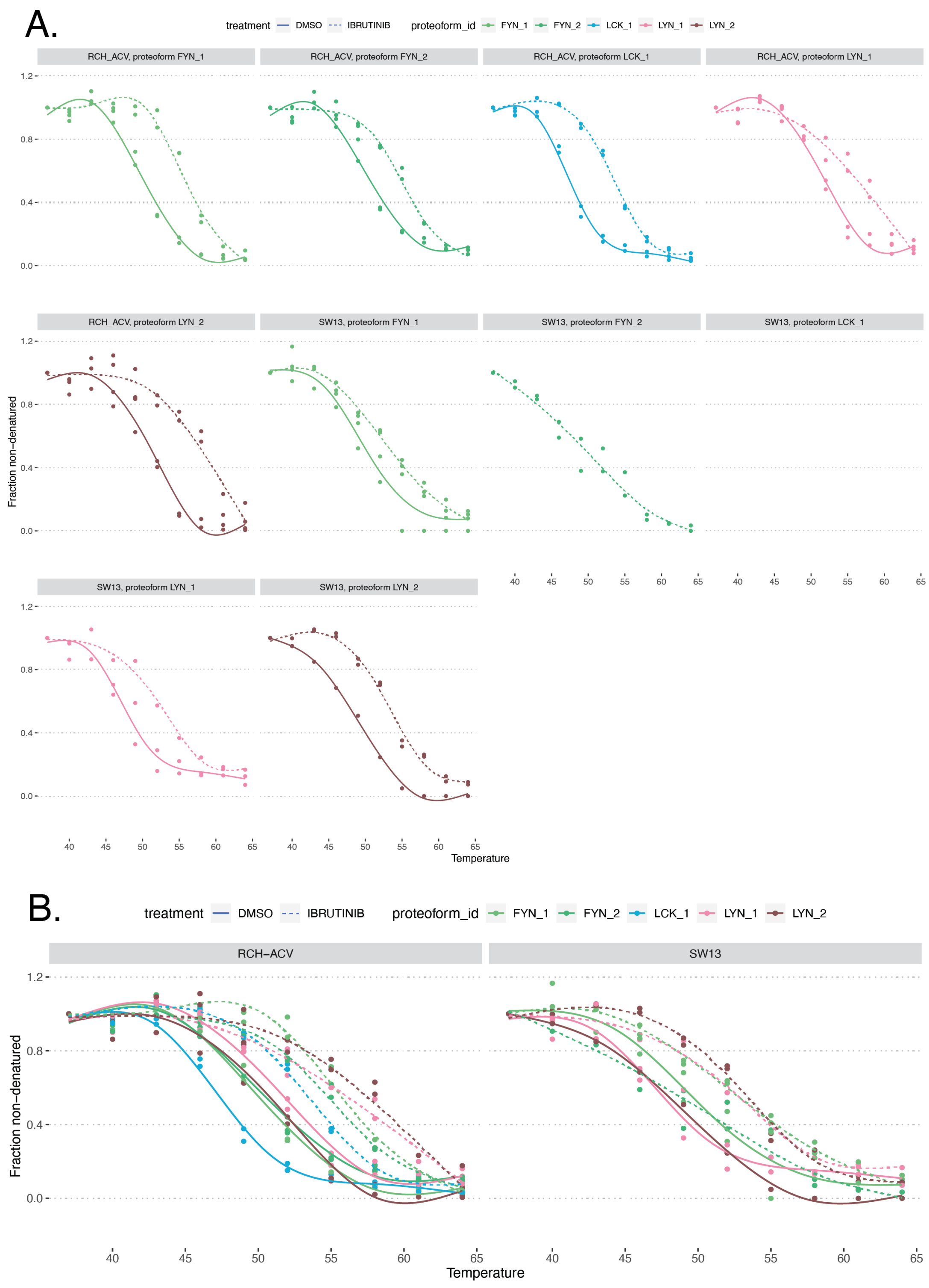

Supplementary Figure 6, Functional proteoform group composition and thermal behavior of the CD20-LCK-LYN-FYN-p75/80 complex: A) All functional proteoform group melting, for the CD20-LCK-LYN-FYN-p75/80 complex. **B)** Same data as (A), plotted in one window per cell line, showing detection per cell line.

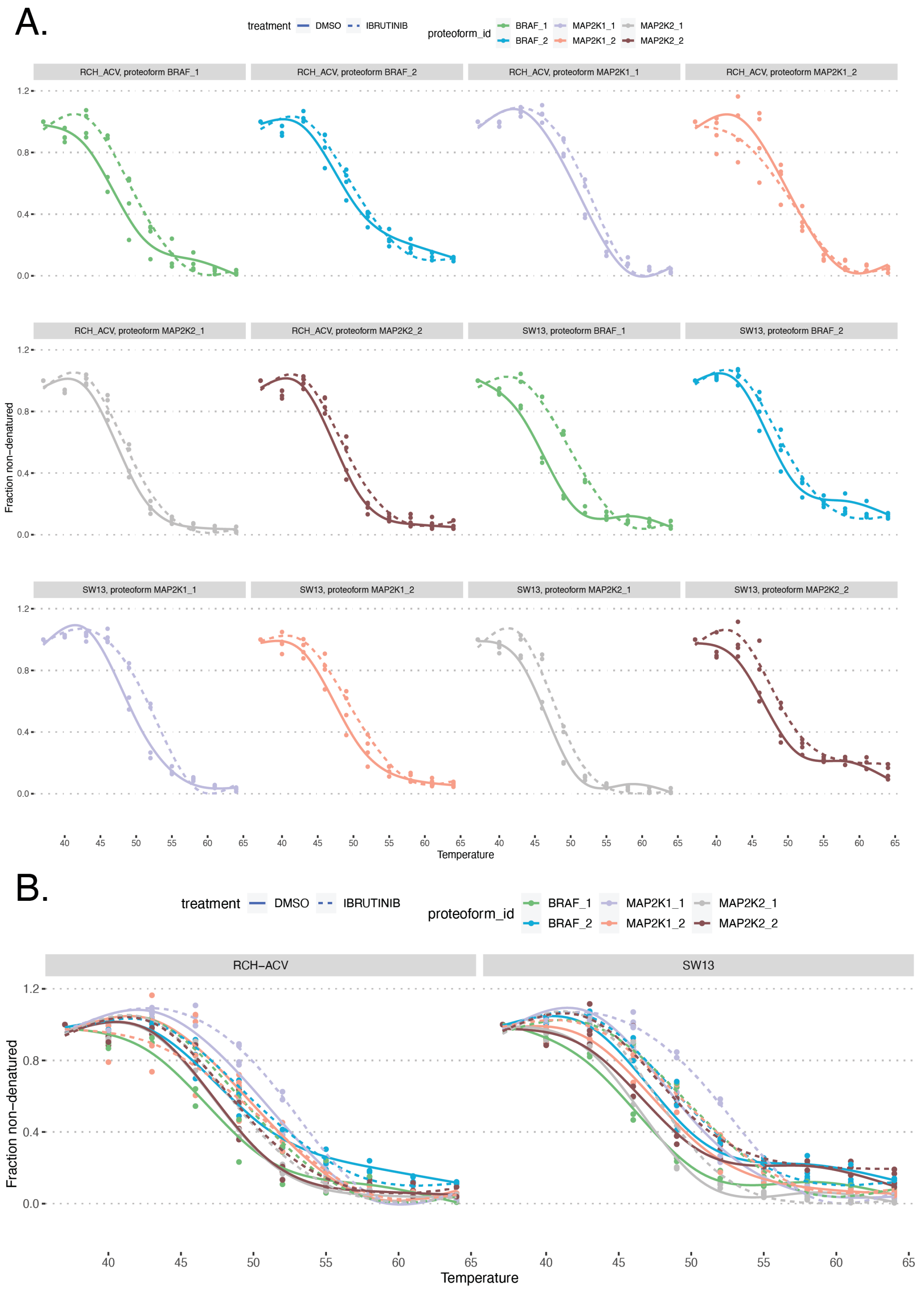

Supplementary Figure 7, Proteoform composition and thermal behavior of the BRAF-MAP2K1-MAP2K2-YWHAE complex: A) All proteoform melting, for the BRAF-MAP2K1-MAP2K2-YWHAE complex. **B)** Same data as (A), plotted in one window per cell line.

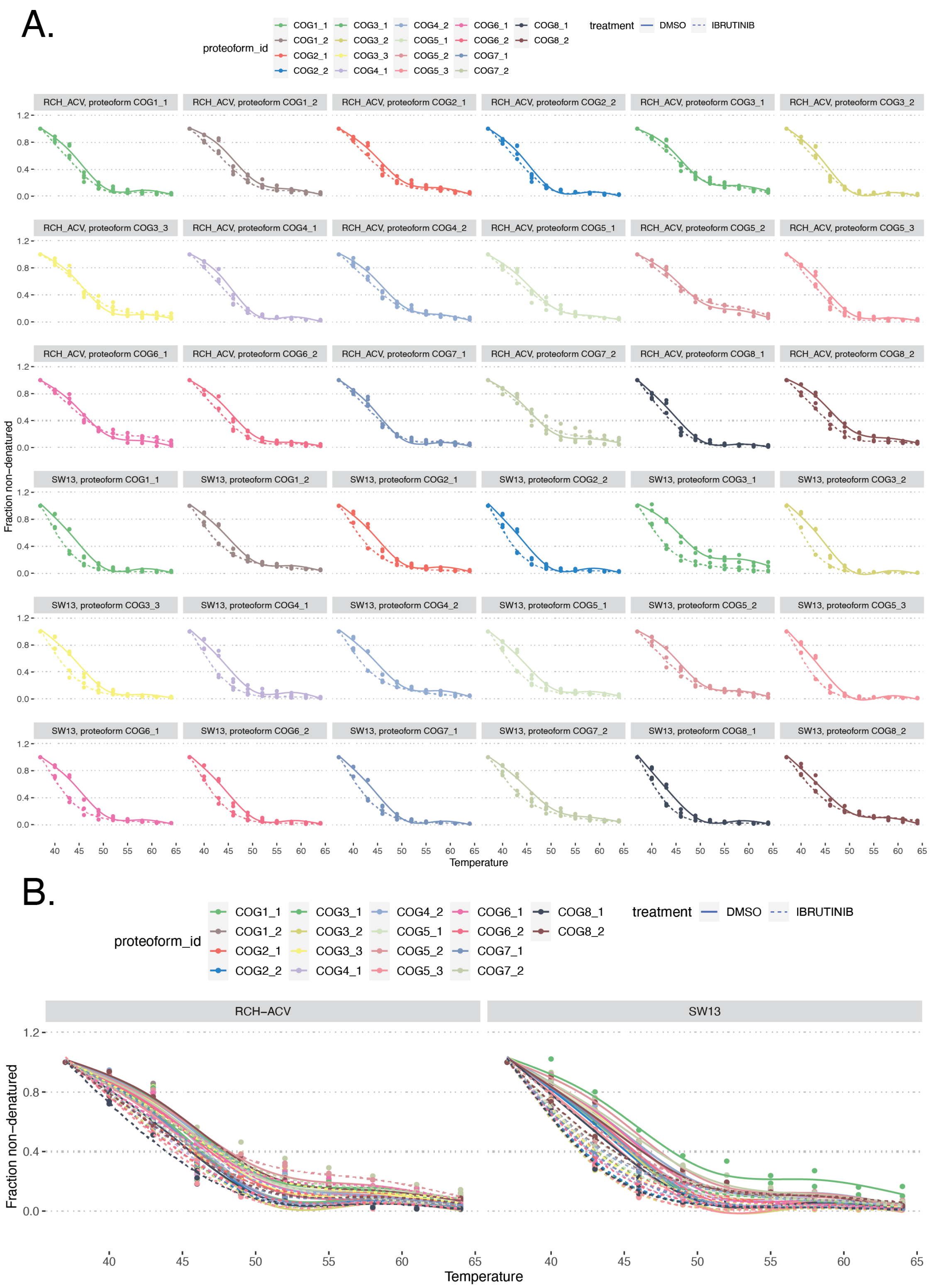

Supplementary Figure 8, Functional proteoform group composition and thermal behavior of the COG complex: A) All functional proteoform group melting, for the COG complex. **B)** Same data as (A), plotted in one window per cell line, showing detection per cell line.

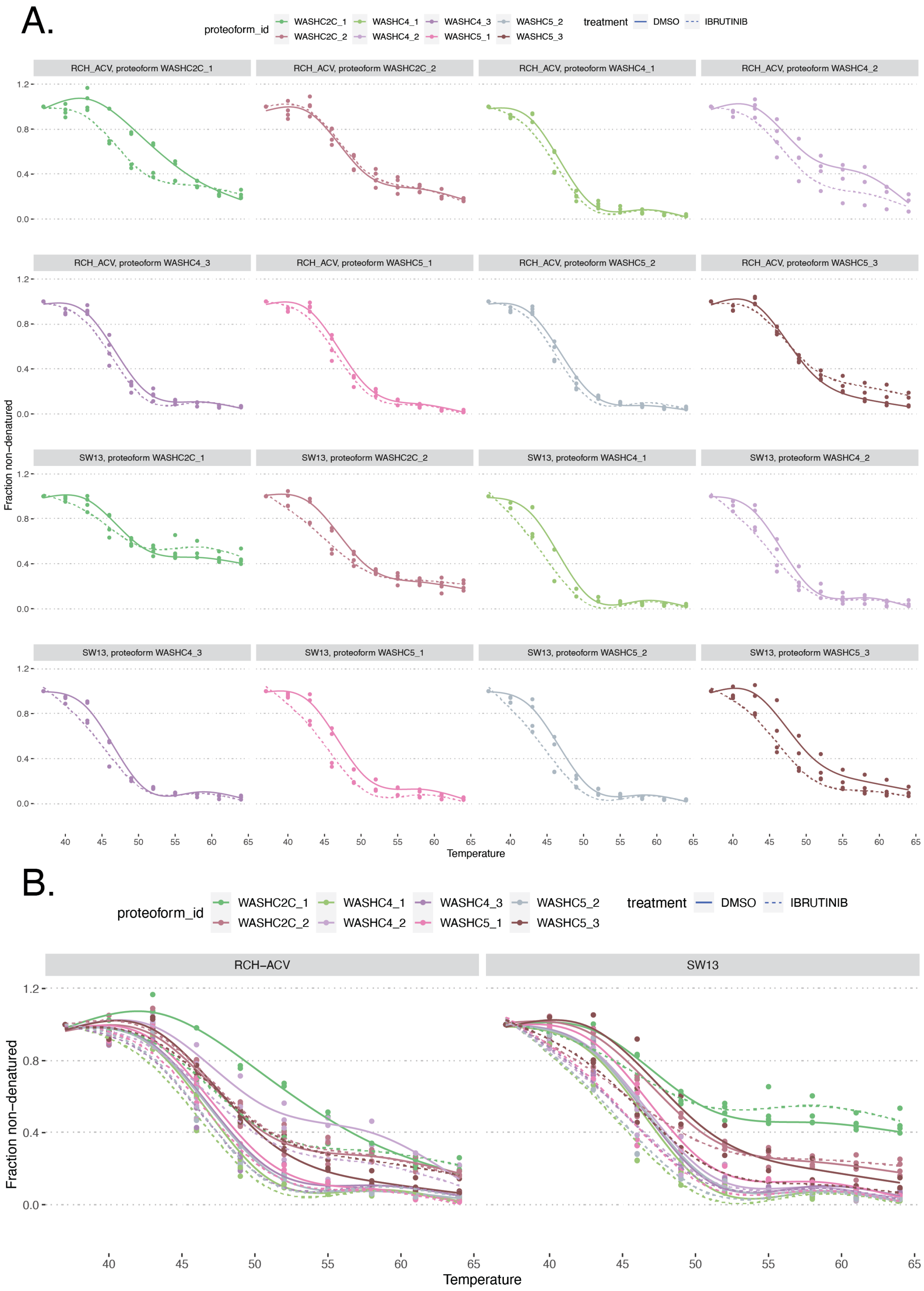

Supplementary Figure 9, Functional proteoform group composition and thermal behavior of the WASH complex: A) All functional proteoform group melting, for the WASH complex. **B)** Same data as (A), plotted in one window per cell line, showing detection per cell line.

A.

Biogrid Interaction Network

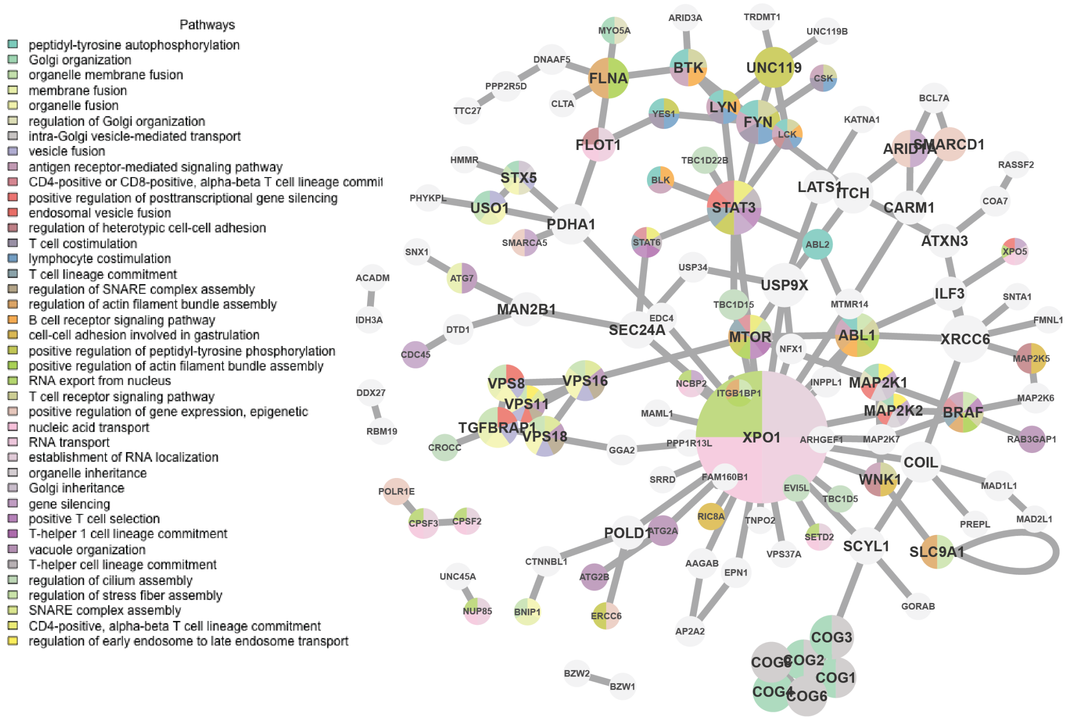

Supplementary Figure 10, Pathway representation in interaction network analysis: A) Network plot showing the GO:biological process pathway distribution, plotted over the sub-network of top NPARC hits and their associations according to the BioGRID interaction database. Nodes are plotted by size according to connectivity, and colored labels indicate membership in an enriched GO:biological process pathway.

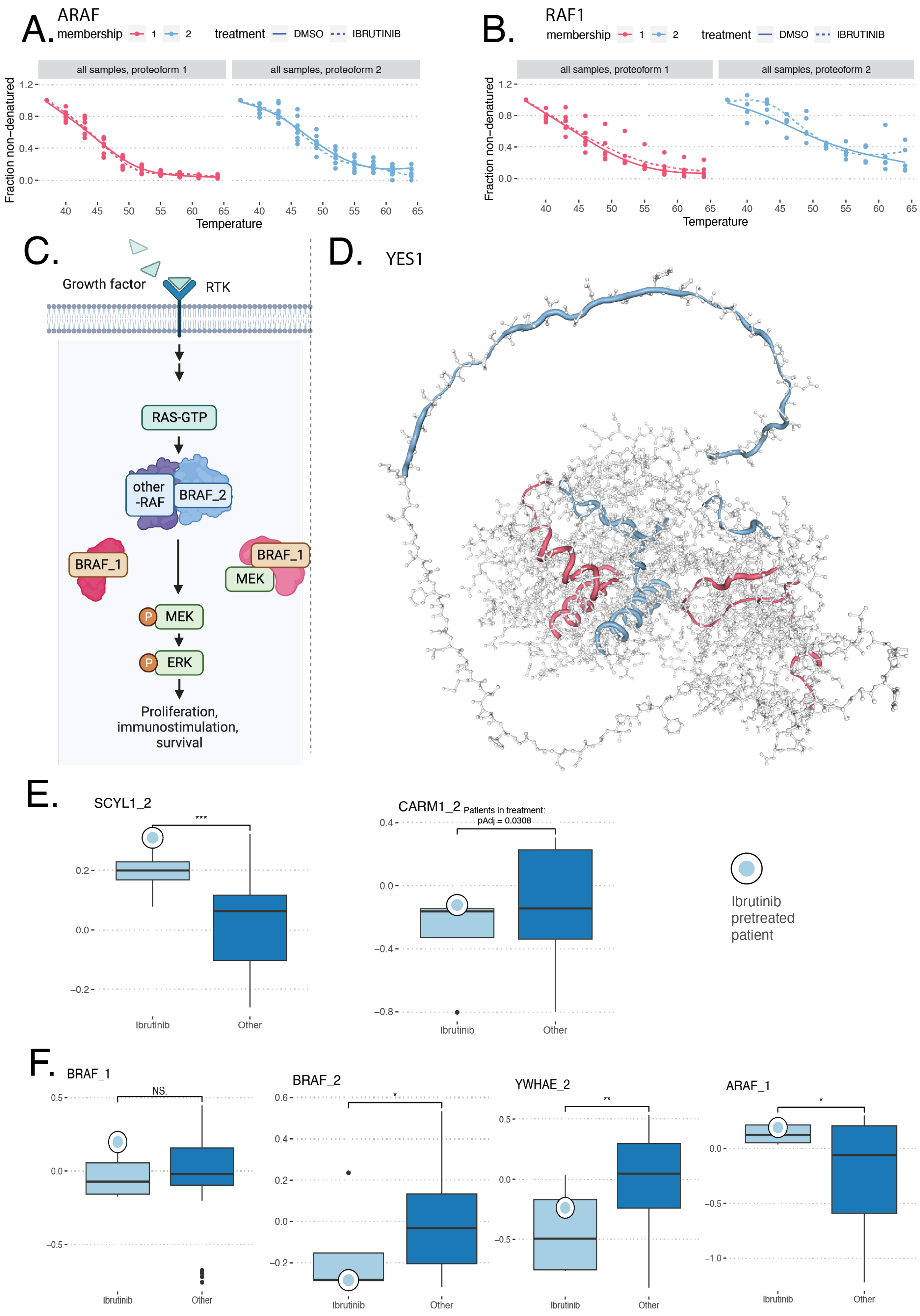

A.
ARAF
membership 1 2
treatment DMSO IBRUTINIB
all samples, proteoform 1
all samples, proteoform 2
Fraction non−denatured
1.2
0.8
0.4
0.0
40 45 50 55 60 65
Temperature
B.
RAF1
membership 1 2
treatment DMSO IBRUTINIB
all samples, proteoform 1
all samples, proteoform 2
Fraction non−denatured
Temperature
C.
Growth factor
RTK
RAS-GTP
other -RAF
BRAF_2
BRAF_1
BRAF_1
MEK
P MEK
P ERK
Proliferation, immunostimulation, survival
D.
YES1
E.
SCYL1_2
***
0.2
0.0
−0.2
Ibrutinib
Other
CARM1_2
Patients in treatment: pAdj = 0.0308
0.4
0.0
−0.4
−0.8
Ibrutinib
Other
Ibrutinib pretreated patient
F.
BRAF_1
NS.
0.5
0.0
−0.5
Ibrutinib
Other
BRAF_2
*
0.6
0.4
0.2
0.0
−0.2
Ibrutinib
Other
YWHAE_2
**
0.5
0.0
−0.5
Ibrutinib
Other
ARAF_1
*
0.0
−0.5
−1.0
Ibrutinib
Other

Supplementary Figure 11, Ibrutinib target refinement: A) Functional proteoform group melting behavior for RAF dimerization partner ARAF, showing data from all cell lines separated by proteoform groups. **B)** Proteoform group melting behavior for the single proteoform group detected for RAF dimerization partner RAF1, showing data from all cell lines. **C)** Illustrated diagram of proposed BRAF proteoform groups. Created with BioRender.com. **D)** Alphafold structure for YES1 generated using the canonical FASTA sequence (sp|P07947|YES1_HUMAN), showing colored tube overlays for peptides colored by their proteoform group assignments. Regions with multiple matched proteoform groups are displayed with blended translucent coloring, and regions without assigned peptides appear as a grey amino acid backbone. **E)** NUMAC complex components SCYL1_2 and CARM1_2, evaluated using Wilcoxon rank sum test with BH correction for multiple comparisons and excluding the ibrutinib pretreated patient in the case of CARM1_2. For SCYL1_2, pAdj = 0.000732. This analysis used n = 3 ibrutinib treated patients with n = 64 controls who received another treatment or were not yet treated. **F)** Vertical from right, four plots show abundance changes linked to BRAF. First in descending order, BRAF_1 and BRAF_2 are compared between treated and untreated patients, pAdj = .442 for BRAF_1, pAdj = .0166 for BRAF_2. Next, YWHAE_2 is shown, representing an indication of a non-RAF interaction, pAdj = .00913. Lastly, ARAF_1 is shown, representing compensatory upregulation of RAF signaling, pAdj = .0403.
