# Supplementary material for: Functional proteoform group deconvolution reveals a broader spectrum of ibrutinib off-targets
Source: Nat Commun. 2025 Feb 25;16:1948. doi: 10.1038/s41467-024-54654-8 (PMC11862126; doi:10.1038/s41467-024-54654-8)
Supplement: Supplementary file 2 — Description of Additional Supplementary Information [file 41467_2024_54654_MOESM2_ESM.docx]

Description of Additional Supplementary Data

Supplementary Data 1: The NPARC analysis, demonstrating thermal differences by ibrutinib treatment. Tests model all data together, or each cell line separately.

**Supplementary Data 2**: List of gene symbols indicated as ibrutinib targets, and the sources that confirm them.

**Supplementary Data 3:** The CORUM complex enrichment over-representation analysis, which analyzed the ibrutinib target list.

**Supplementary Data 4**: The Biogrid network topology analysis of functional proteoform group hits from the ibrutinib target list.

**Supplementary Data 5:** MS pull down experiment results table, showing intensity values for ibrutinib and DMSO controls at 100nM and 20uM.

**Supplementary Data 6:** Meta data of the clinical CLL patient cohort.

**Supplementary Data 7:** Comparison of functional proteoform group abundance in ibrutnib treated CLL patient samples, compared to patients who received other therapy or were untreated.
